# SciKit Digital Health: Python Package for Streamlined Wearable Inertial Sensor Data Processing

**DOI:** 10.2196/36762

**Published:** 2022-04-21

**Authors:** Lukas Adamowicz, Yiorgos Christakis, Matthew D Czech, Tomasz Adamusiak

**Affiliations:** 1 Digital Medicine and Translational Imaging Pfizer Inc Cambridge, MA United States

**Keywords:** wearable sensors, digital medicine, gait analysis, human movement analysis, digital biomarkers, uHealth, wearable, sensor, gait, movement, mobility, physical activity, sleep, Python, coding, open source, software package, algorithm, machine learning, data science, computer programming

## Abstract

Wearable inertial sensors are providing enhanced insight into patient mobility and health. Significant research efforts have focused on wearable algorithm design and deployment in both research and clinical settings; however, open-source, general-purpose software tools for processing various activities of daily living are relatively scarce. Furthermore, few studies include code for replication or off-the-shelf software packages. In this work, we introduce SciKit Digital Health (SKDH), a Python software package (Python Software Foundation) containing various algorithms for deriving clinical features of gait, sit to stand, physical activity, and sleep, wrapped in an easily extensible framework. SKDH combines data ingestion, preprocessing, and data analysis methods geared toward modern data science workflows and streamlines the generation of digital endpoints in “good practice” environments by combining all the necessary data processing steps in a single pipeline. Our package simplifies the construction of new data processing pipelines and promotes reproducibility by following a convention over configuration approach, standardizing most settings on physiologically reasonable defaults in healthy adult populations or those with mild impairment. SKDH is open source, as well as free to use and extend under a permissive Massachusetts Institute of Technology license, and is available from GitHub (PfizerRD/scikit-digital-health), the Python Package Index, and the conda-forge channel of Anaconda.

## Introduction

Wearable inertial sensors have enabled huge leaps forward in the ability to quantify and derive actionable insights from patient mobility and at-home health. Algorithm development and deployment in both research and clinical studies have been a focus of many research efforts. For example, gait monitoring using wearables has evolved from algorithm design using minimal sensors for the purpose of minimizing patient burden [1-5] to at-home deployment and remote monitoring of free-living activity [6-8]. Remote patient monitoring has a high intrinsic value, as previous work has suggested. At-home values may be less influenced by observer effects [8,9] and may facilitate enhanced group differentiation [8-10].

While lumbar-mounted sensors are appealing for capturing bilateral gait and other lower body activities such as sit-to-stand transfers, wrist sensors are also desirable as they can be integrated into watches or watch-like packages and offer lower subject burden. Sleep and physical activity monitoring, which typically use a wrist-based sensor, are also among extensively researched areas [11-18]. Sleep and physical activity research have been aided by the availability of an open-source, freely available code package, GGIR [19]. GGIR is a collection of algorithms for activity and sleep research, written in R, and includes code to ingest, calibrate, and detect sleep and activity level from raw acceleration data. GGIR allows researchers to study patient symptoms with limited programming expertise and has been evaluated in over 90 peer-reviewed journal publications [19].

The availability of GGIR is in stark contrast with many other published works in this area. Relatively few works include any code for experiment replication, and even fewer include easy-to-use or “off-the-shelf” code packages, despite the ease of sharing through public code repositories such as GitHub. Our group has made an effort to release several implementations from existing research or new algorithms for gait [8], sleep [20], and sit to stand [10]. However, open-source packages to date are fairly disparate and require additional steps for data ingestion and preprocessing. Other options include the Digital Biomarker Discovery Pipeline [21], a partial set of tools with the goal of enhancing data inspection, cleaning, and processing to enable digital biomarker discovery. However, it is composed of separate modules with iPython notebooks instead of Python libraries, and currently the project seems dormant (the last update was on November 3, 2020). Open-source GENEActiv R macros also exist, even though they are specific to GENEActiv files and would require custom modification to ingest data from other devices.

The lack of open-source, general-purpose algorithms for the processing of the various base activities of daily living is a significant gap in the field. By addressing this limitation, we hope to advance human activity recognition research in two important ways: (1) lowering the requirements for analyzing longitudinal data and (2) providing a baseline set of algorithm implementations for the community. Additionally, given the ease of sharing code, we hope to encourage the practice of sharing code with publications—an approach that should be adopted from other areas such as machine or deep learning research and encouraged by the National Institutes of Health.

In this paper, we present a new Python package, SciKit Digital Health (SKDH), to address the lack of open-source, general-purpose algorithms for monitoring digital health. SKDH contains algorithms for various measures of human activity recognition and streamlines the data ingestion, preprocessing, and data analysis steps. While the underlying algorithms themselves are not necessarily novel work, the novelty and utility of this work is the collection of common mobility and activity algorithms under a common framework that is being released open source. SKDH aims to address the shortcomings in available, existing codebases by (1) being easily usable with minimal interaction required from end users; (2) being tightly integrated so that different processing modules can be easily chained together, allowing multiple preprocessing and analysis steps in the same pipeline; and (3) being free and open source.

## Methods

### SciKit Digital Health

SKDH is a Python 3 package that contains algorithms for gait, sit to stand, activity level, and sleep. Additionally, it contains various preprocessing methods such as accelerometer calibration; wear detection; and binary file data readers for the GENEActiv, Axivity, and ActiGraph sensors. Individual algorithms or steps are built around an extensible process class (“BaseProcess”), which are chained together as needed in a pipeline structure. The BaseProcess class abstracts various setup tasks and standardized functions that allow for subclasses to function properly and in sequence in the SKDH framework. This allows the end user to easily link steps together, as shown in Textbox 1.

SKDH also contains various common utility functions (eg, moving mean, standard deviation with arbitrary window length, and skip values) and a suite of features for signal processing and feature generation for machine learning, written in C or Fortran, to reduce computation time (Table 1).

**Figure 1:**
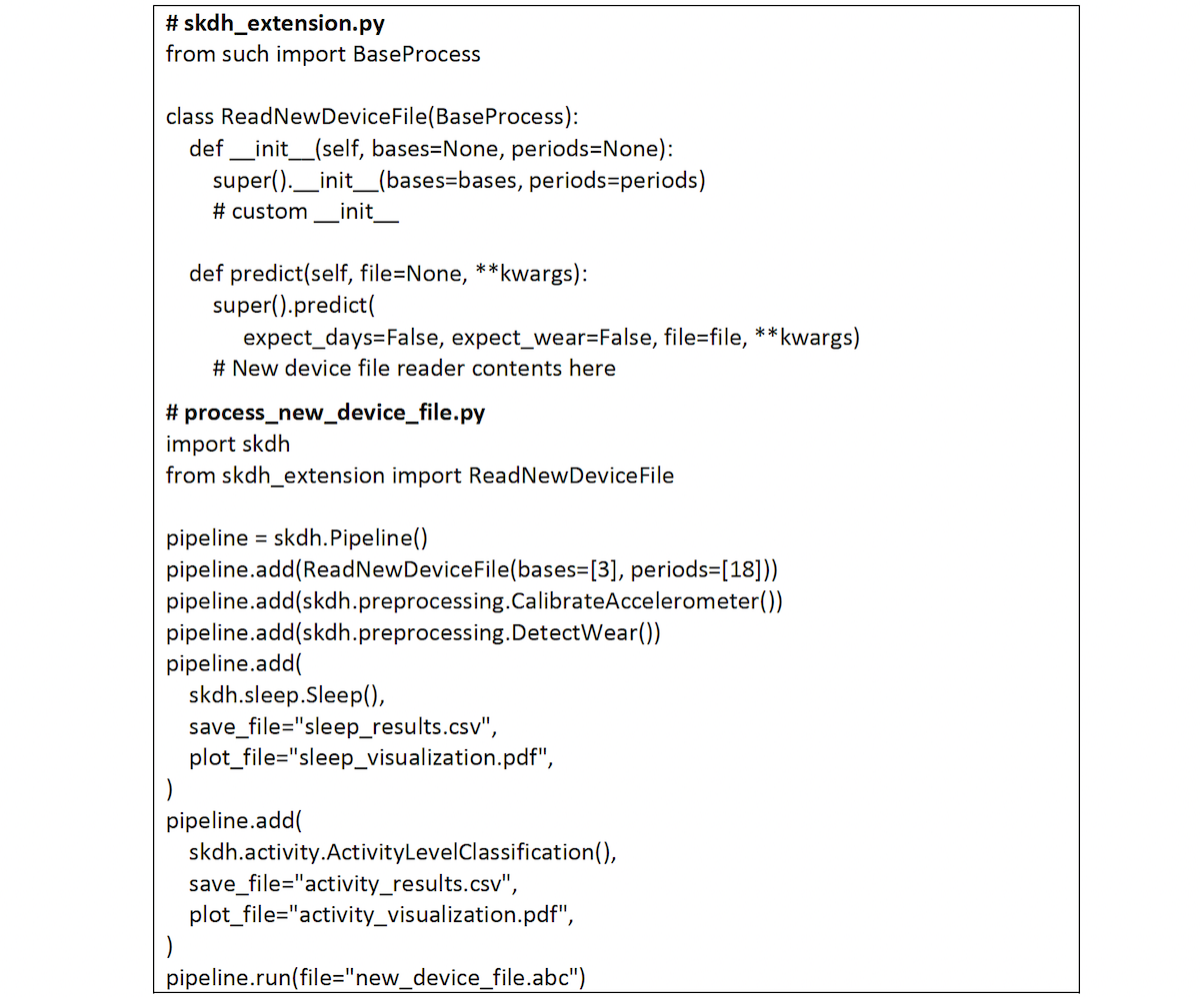
A custom class for reading a file from a new device is first created as a subclass of SciKit Digital Health's (SKDH) "BaseProcess" that allows it to be easily inserted into a SKDH pipeline. Note that SKDH will automatically save results from the default sleep and activity analyses to the specified files.

A more comprehensive example that shows how SKDH base classes can be extended and easily integrated into an SKDH pipeline is shown in Figure 1.

Additionally, to simulate a realistic processing scenario, the data was windowed over 3-second windows (150 samples) with 50% overlap, and the computation was run again.

**Example script that will (1) import data from a GENEActiv bin file, (2) calibrate the accelerometer so that still periods measure 1 g, and (3) run gait processing to generate gait endpoints.**

import skdh

pipeline=skdh.Pipeline()

pipeline.add(skdh.io.ReadBin())

pipeline.add(skdh.preprocessing.CalibrateAccelerometer())

pipeline.add(skdh.gait.Gait())

pipeline.run(file=“example_geneactiv_file.bin”)

**Table 1:** Mean (SD) processing times in milliseconds on a representative array of random^a^ data.

Feature	100,000 3 array^b^			Windowed: 3s, 50% overlap^c^		
	Original^d^ (ms), mean (SD)	SciKit Digital Health (ms), mean (SD)	Factor	Original (ms), mean (SD)	SKDH (ms), mean (SD)	Factor
Signal entropy	12.7 (0.31)	1.53 (0.03)	8.3	3008 (88.7)	3.89 (0.21)	792
Jerk metric	22.6 (1.86)	0.05 (0.02)	45	2720 (80.8)	0.97 (0.07)	2810
Spectral arc length	1005 (24.7)	197 (3.70)	5.3	3340 (102)	115 (3.74)	29

^a^NumPy.random.default_rng().standard normal.

^b^Produces 3 values for the feature.

^c^1332 resulting windows. Original runs 3 separate data frames (shape (150, 1332)), one for each XYZ axis. SKDH features run on, full shape (1332, 150, 3) array.

^d^Implemented with NumPy for Pandas input.

### Gait

The gait algorithm uses the inverted pendulum model of gait to extract bilateral gait endpoints from acceleration data collected from a lumbar-mounted wearable inertial sensor [1-4]. In general, gait bouts during free-living data are first detected using a gradient boosted tree classifier [22]. For in-lab data in which the time periods of gait are known, the gait classification step can be skipped. Wavelet transforms are then used to detect initial and final contact events for each foot from the vertical acceleration signal [4]. With these contact events, all temporal endpoints (eg, stride time, double support, etc) are computed. In order to obtain spatial metrics (eg, stride length, gait speed, etc), an inverted pendulum model [1] is used, requiring only the participant’s height in addition to the vertical acceleration signal.

The implementation is very similar to that of our previously released GaitPy package [8], updated to fit into the SKDH architecture, with a few key algorithm additions and updates. Notably, the classifier for gait bouts during at-home periods has been updated, using the training data from 4 additional studies to gain a better breadth of nongait activities. These studies are “the daily life activities” [23], “the long term movement monitoring database” [24], “the University of Southern California human activity dataset” [25], and “a Parkinson’s disease study” [26].

In the original GaitPy wavelet transform implementation, a fixed scale was used. However, recent research shows that the scale can be better optimized by matching it to the step frequency [5], and this relationship was added as an optional toggle. Finally, additional asymmetry endpoints were added, including but not limited to the gait symmetry index [27-29], step and stride regularity [3,28], and intrastep and intrastride covariance [28].

### Sit to Stand

The sit-to-stand algorithm is identical to what was released in Sit2StandPy [10], though integrated into the SKDH framework. It uses acceleration data from a lumbar-mounted device to identify sit-to-stand transfers in both in-lab and free-living environments. The sit-to-stand algorithm is a heuristic algorithm, which functions by identifying possible sit-to-stand locations using a wavelet transform and acceleration filtering. With possible locations identified, a series of quality checks and rules are imposed to determine whether the transfer is valid or not. Validation for the algorithm was previously presented using data from patients with Parkinson’s disease and healthy adults [10].

### Sleep

The sleep algorithm in SKDH was originally presented in the Python package, SleepPy [20-30], and here, it was adapted into the SKDH framework. This algorithm was originally based on the one implemented in GGIR [31]. It is intended for use on the acceleration data from the wrist, even though it will also take advantage of near-body temperature data, if available, to significantly improve sleep-specific, on-body detection. The algorithm first determines 1 sleep opportunity window per day (noon to noon) using a series of moving mean and median filters. During this period, bouts of sleep and wake are determined by computing the activity index [32] of the acceleration data and then applying a heuristic scoring algorithm [33]. Sleep endpoints are then calculated, including but not limited to wake after sleep onset, total sleep time, as well as sleep and wake transition probabilities [34,35]. If desired, a per-day sleep plot can be produced as well for visual inspection.

### Activity

**Figure 2:**
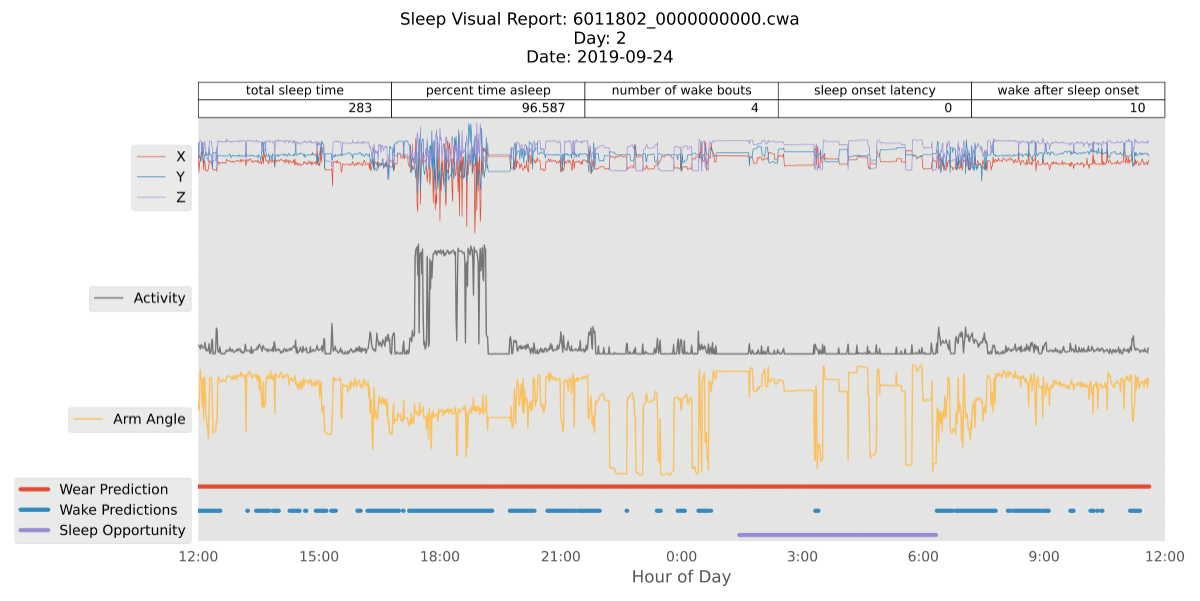
A sample sleep plot as produced by SciKit Digital Health, showing a single night from test data.

The activity algorithm seeks to provide similar outputs to previously published research [15,16,18] such as time spent in sedentary, light, moderate, or vigorous activity levels. Wrist-based triaxial acceleration is windowed into 5-second blocks, and the mean is taken. By default, the value of gravity is subtracted to obtain the Euclidean norm minus one (ENMO). These ENMO values are then used to threshold into different activity levels with different provided base options derived from the literature [13,15,16,18]. These periods of time in different activity levels can also be rescored to obtain bouts of consistent activity level [14,18,36]. Finally, recent work has also proposed alternative methods of accessing activity level by quantifying the decline in the time spent in increasing activity magnitude [17]; this analysis is also included in the activity endpoints. Similar to the sleep plot, a per-day activity plot can be saved if desired, showing the acceleration, activity, activity level, and wear traces, as seen in Figure 2.

## Research Applications

Use cases for research applications are widely varied and cover a broad spectrum of research topics in the relevant fields. First and foremost, SKDH provides a quick and easy-to-use tool to generate activity and mobility endpoints with limited adjustment and setup required on the part of researchers or clinicians. Since default parameter values for algorithms are set to physiological defaults, SKDH would provide an “off-the-shelf” experience when the research goal is endpoint assessment or comparison.

However, the adjustable algorithm parameters also allow for a more nuanced approach if the research goal is instead exploring the algorithms themselves. Along with this, as the code is open source, researchers are also able to use SKDH as a starting point and add functionality or improvements as they need for their work.

These utilization strategies for SKDH in research on gait, sit to stand, activity, and sleep lead to a broad range of applications for SKDH in research. On top of this, many of the additional utility or feature generation capabilities present in SKDH are useful outside the context of these activities as well, for initial data exploration or even just for ingestion of data from sensor binary file formats.

## Validation

Validation of algorithm implementation is critical to ensure that the generated results match the expected values and provide actionable insight. For SKDH, validation is an ongoing effort with the different modules having different levels of validation, even though all the individual algorithms were validated in their original publications. Validation for the sit-to-stand module included in SKDH was presented previously [10], and the algorithm implementation remained exactly the same. The gait and the sleep modules had previous implementations validated in previous publications (gait in a study by Czech et al [8], and sleep in a study by Mahadevan et al [20]), even though there are implementation differences and algorithm additions in SKDH. Internal validation of the gait module showed a higher agreement and tighter ranges of intraclass correlation coefficients compared with the previous versions of the gait implementation (results not shown). The activity module has also shown excellent agreement in internal comparisons to GENEActiv macros and GGIR (results not shown).

## Abbreviations

ENMO: Euclidean norm minus one
SKDH: Scikit Digital Health

## Data Availability

The source code is available under the Massachusetts Institute of Technology license from GitHub (PfizerRD/scikit-digital-health). Installable packages will be available on the Python Package Index and the conda-forge channel of Anaconda.
